# Psychomotor development of preterm infants aged 6 to 12 months

**DOI:** 10.1590/S1516-31802012000500006

**Published:** 2012-11-13

**Authors:** Sophie Helena Eickmann, Natália Ferraz de Araújo Malkes, Marília de Carvalho Lima

**Affiliations:** I MD, PhD. Adjunct Professor, Department of Maternal and Child Health, Universidade Federal de Pernambuco (UFPE), Recife, Pernambuco, Brazil.; II MSc. Physiotherapist and Assistant Professor, Physiotherapy Course, Associação Caruaruense de Ensino Superior (ASCES), Caruaru, Pernambuco, Brazil.; III MD, PhD. Adjunct Professor, Department of Maternal and Child Health, Universidade Federal de Pernambuco (UFPE), Recife, Pernambuco, Brazil.

**Keywords:** Infant, premature, Biological factors, Risk factors, Child development, Language development, Prematuro, Fatores biológicos, Fatores de risco, Desenvolvimento infantil, Desenvolvimento da linguagem

## Abstract

**CONTEXT AND OBJECTIVE:**

The immaturity of preterm infants’ organ systems may lead to difficulties in adapting to different environmental stimuli. The aim was to compare the psychomotor development of preterm infants (with corrected age) and term infants aged 6 to 12 months and to investigate associated factors.

**DESIGN AND SETTING:**

Cross-sectional analytical study conducted at Hospital das Clínicas, Universidade Federal de Pernambuco.

**METHODS:**

The sample consisted of 135 infants (45 preterm and 90 full-term) aged 6 to 12 months. Neuropsychomotor development was assessed using the Bayley III cognitive, language and motor subscales. Biological, socioeconomic and demographic data were gathered from medical records and through interviews with mothers.

**RESULTS:**

The mean cognitive, language and motor indices were within the range of normality for the sample as a whole. No significant difference in the development of infants born preterm and full-term was observed, except for expressive communication, in which preterm infants presented a lower index. Motor development was influenced by biological factors, and the poorest performances were observed in male infants; birth weight birth weight < 1500 g; Apgar score at five minutes ≤ 7; weight-, length- and head circumference-for-age < -1 Z-score; and exclusively breastfeeding for ≤ two months.

**CONCLUSIONS:**

Prematurity did not influence the psychomotor development of infants in this study population. Motor development was the most affected domain in the sample as a whole, especially due to biological factors. Investigations on child neuropsychomotor development should try to identify many determinant factors because of its multifactorial nature.

## RESUMO

**CONTEXTO E OBJETIVO:**

A imaturidade dos sistemas orgânicos das crianças nascidas prematuras pode levar a dificuldades de adaptação a diferentes estímulos do ambiente. O objetivo foi comparar o desenvolvimento neuropsicomotor de lactentes nascidos pré-termo (com idade corrigida) e no termo com idade entre 6 e 12 meses e investigar seus fatores associados.

**TIPO DE ESTUDO E LOCAL:**

Estudo transversal analítico realizado no Hospital das Clínicas, Universidade Federal de Pernambuco.

**MÉTODOS:**

A amostra foi constituída de 135 crianças (45 pré-termo e 90 no termo) com idade entre 6 a 12 meses. O desenvolvimento neuropsicomotor foi avaliado pelas subescalas cognitiva, linguagem e motora da Bayley III. Dados biológicos, socioeconômicos e demográficos foram coletados de prontuários médicos e por entrevistas com as mães.

**RESULTADOS:**

A média dos índices cognitivo, de linguagem e motor estava dentro da faixa de normalidade para a amostra como um todo. Não foi observada diferença significativa entre o desenvolvimento de crianças nascidas pré-termo e no termo, com exceção para a comunicação expressiva, na qual os prematuros apresentaram menor índice. O desenvolvimento motor foi influenciado por fatores biológicos, e os piores desempenhos foram observados em crianças do sexo masculino, com peso ao nascer < 1500 g; índice de Apgar aos cinco minutos ≤ 7; peso, comprimento e circunferência da cabeça-para-idade < -1 escore Z; e amamentação exclusiva por ≤ dois meses.

**CONCLUSÕES:**

A prematuridade não influenciou o desenvolvimento psicomotor das crianças nessa população. O desenvolvimento motor foi o domínio mais afetado na amostra como um todo, especialmente devido a fatores biológicos. Investigações sobre o desenvolvimento neuropsicomotor infantil devem tentar identificar inúmeros fatores determinantes devido a sua natureza multifatorial.

## INTRODUCTION

Concern about the psychomotor development of preterm infants has grown over recent decades because of increasing infant survival rates together with healthcare professionals’ commitment towards improving the quality of life of this population. Psychomotor development occurs through a process of complex transformations, including growth, maturation, learning and psychosocial factors.^1^ It is characterized by a set of functional domains including cognition, language, gross and fine motor skills, sensory functions and socioemotional development acquired over time, through neural maturation and environmental experiences.^2^^,^^3^

Infant development is influenced by several different factors, including biological determinants relating to pregnancy and birth, which are more closely linked to development outcomes during the first year of life;^4^^,^^5^ and environmental factors, such as family socioeconomic, demographic and cultural characteristics, which gain enhanced influence as postnatal age increases.^4^^,^^6^

Current studies emphasize that prematurity is one of the major biological risk factors for development.^4^^,^^5^^,^^6^^,^^7^^,^^8^ Immature organ systems are common in premature birth, and may lead to difficulties in adapting to different environmental stimuli. This biological vulnerability may increase the infant’s chances of presenting disorders during various stages of development later in life.^5^^,^^9^^,^^10^^,^^11^^,^^12^ However, because of this complex interaction between biological and environmental factors, predicting the course of development within this population is not an easy task.

Early identification of developmental problems is an intricate task, and detection of any deviation requires systematic assessments, especially during the first year of life, when the developmental process is more dynamic.^9^ The sooner the problems are detected, the sooner it will be that an early multidisciplinary approach can begin, involving professionals from different fields like social services, health and education. In this manner, a wide range of support can be provided for both the infants and their families.^10^^,^^13^ Most studies that assess the development of preterm infants are conducted in developed countries, and therefore, insufficient knowledge is available regarding the impact of preterm birth on infant development in populations living under adverse socioeconomic conditions.

## OBJECTIVE

The aim was to compare the psychomotor development of preterm infants (with corrected age) and term infants aged 6 to 12 months and to investigate its associated factors in a population of low socioeconomic status.

## METHODS

### Study design, setting and sample

This was a cross-sectional analytical study conducted at the High-Risk Infant Follow-up Clinic and the Well Baby Clinic at Hospital das Clínicas, Universidade Federal de Pernambuco (UFPE). The first of these clinics admits infants born at the maternity hospital, which is a high-risk pregnancy referral unit. The number of deliveries in 2008 was 1390, of which 17% were preterm, composing a total of 243 live-birth preterm infants. Of these 42 died, and thus, 201 babies were discharged from hospital. Therefore, it was expected that 84 preterm babies would be admitted to the High-Risk Infant Follow-up Clinic during the five month data-gathering period. However, only 45 of them met the inclusion criteria or attended this clinic. The main reason why this follow-up clinic was not used in the cases of the other infants was that they lived far away from Hospital das Clínicas, which is located in Recife, the capital of the state of Pernambuco, considering that this hospital also admits high-risk pregnant women from different municipalities in the interior of the state.

The exclusion criterion was the presence of congenital infections and/or malformations and genetic syndromes. All preterm infants registered at the High-Risk Infant Follow-up Clinic during the study period were recruited.

The sampling procedure for selecting the control group consisted of enrolling the next two infants born at term who attended the Well Baby Clinic, after each preterm infant had been recruited. In this manner, the study sample was made up of 135 infants aged 6 to 12 months: 45 born preterm and 90 born at full-term.

### Sample size calculation

A power analysis calculation was conducted using the software G*Power 3 to compare pairs of independent sample means. Assuming an a error of 0.05, a study power of 80% and an effect size of 0.40 for detecting a difference in mean developmental score between the groups, together with the infant ratio of 2:1, the total sample requirement was estimated to be 140 infants (47 preterm and 93 term infants).

### Data gathering

This study was undertaken from March to July 2008, after conducting a pilot study to ensure consistency and standardization of the techniques and research tools. Assessments of nutritional status and psychomotor development among the preterm infants was done with correction of the chronological age, by subtracting the number of days required for completing 40 weeks of gestation that remained at the time of birth, from the chronological age.^14^

#### Clinical and anthropometric assessment at birth

Data on neonatal morbidity, anthropometric assessments and gestational ages were obtained from hospital records and registered onto precoded forms. The clinical data consisted of the Apgar score at five minutes after birth, to assess neonatal vitality, and occurrences of meningitis, seizures, intracranial hemorrhage and oxygen use.

Birth weight was recorded using digital electronic scales, and head circumference was measured with a flexible tape measure passing across the eyebrow and round to the occipital protuberance. Gestational age was estimated by means of the Capurro method, and the Ballard method was used for infants weighing less than 1500 g. Size for gestational age was classified using the intrauterine growth curve of Lubchenco et al.^15^

#### Socioeconomic characteristics and breastfeeding practice

Socioeconomic and demographic data (family income, education level, maternal age, size of family and number of infants under five at home) and type and duration of breastfeeding were obtained through interviews with the mothers, using a form with precoded closed questions.

Exclusive breastfeeding was defined as situations in which the infant received only breast milk, either directly or extracted from the breast, with no solid food or liquid, except for drops consisting of vitamins, minerals or medicines, during the 24 hours prior to the interview.

#### Anthropometric and nutritional assessments

The anthropometric assessment consisted of recording the weight, length and head circumference in accordance with standard procedures.^16^ This was done by a previously trained research assistant. Weights were recorded on a Filizola BP Baby electronic scale with a maximum capacity of 15 kg, and lengths were measured using a wooden measuring stick with a range of 130 cm in subdivisions of 0.1 cm. Head circumference was measured using an ISP plastic tape measure of 150 cm in length, with subdivisions of 0.1 cm.

Nutritional status was assessed in terms of weight-for-age, length-for-age and head circumference-for-age, expressed as mean Z scores, using the World Health Organization (WHO) standard references (WHO Anthro 2006, version 2.0). Infants were classified as undernourished or nutritionally at risk if the index was lower than -1 Z-score.^16^

#### Developmental tests

The cognitive, language (receptive and expressive communication) and motor (fine and gross skill) subtests of the Bayley Scales of Infant and Toddler Development III^17^ were applied by an experienced tester in the presence of the infant’s mother at the hospital. Any child who was ill at the time of the test was treated and the test was postponed. To interpret the results, the total score (raw score) obtained in each subtest was converted into a balanced score for different age groups and then subsequently converted into composite scores. Each composite score had a mean of 100 points and a standard deviation of 15 points, and a mean balanced score of 10 points with a standard deviation of 3 points.^17^

### Data processing and analysis

Data entry and statistical analysis were carried out using Epi-Info version 6.04d (CDC, Atlanta, United States). Double data entry was used and validated in order to minimize any possible typing errors. Psychomotor developmental scores were treated as continuous variables and their associations with the explanatory variables were assessed as differences between means, using the Student t test or Analysis of Variance (ANOVA) as significance tests in bivariate analyses. The chi-square test or Fisher’s exact test was used when indicated to assess associations between categorical variables. P values £ 0.05 were taken to be significant.

### Ethical permission

This study was approved by the Research Ethics Committee at the Health Sciences Center of UFPE, and was registered under no. 413/07. Informed consent was obtained from parents or guardians for their children to take part in the study and for the developmental tests and anthropometric measurements to be made.

## RESULTS

This group of preterm infants could be considered to present moderate risk, since the variation in gestational age was from 25 to 36 weeks, and only three infants had gestational age less than or equal to 30 weeks. Moreover, the mean birth weight was 1762 g and the lowest Apgar score was 4, thus showing that the birth vitality was moderate to good. A relatively low frequency of perinatal and postnatal morbidity was observed, given that 7% had meningitis, 13% had intracranial hemorrhage grades I to II and none had seizures. Among the 38 infants who required oxygen therapy, 29% underwent assisted mechanical ventilation (median duration of four days) and 82% used continuous positive airway pressure (CPAP), with a median duration of one day.

Table 1 shows that the distribution according to sex and age at the time of assessment was homogeneous between the gestational age groups. A significantly higher percentage of the preterm infants presented an Apgar score at 5 minutes that was less than or equal to 7. There was no difference between the two groups in terms of family socioeconomic and demographic characteristics. Although three-quarters of the families lived below the poverty line (monthly income of up to half a minimum salary per person), the maternal educational level was moderate and all mothers had attended school. The preterm infants presented significantly higher percentages of weight-for-age and length-for-age < -1 Z-score at the time of assessment.

**Table 1.** Selected characteristics of infants born preterm and full-term

Variables	Preterm (n = 45)		Full-term (n = 90)		P
At birth	Mean	(SD)	Mean	(SD)	
Gestational age (weeks)	33.4	(2.4)	39.6	(1.0)	< 0.001
Birth weight (g)	1762	(481)	3425	(446)	< 0.001
Head circumference (cm)^*^	30.0	(2.3)	34.8	(1.3)	< 0.001
Apgar score at 5 minutes^†^ [n (%)]					
4-7	7	(15.6)	1	(1.2)	0.003^‡^
8-10	38	(84.4)	83	(98.8)	
Size for gestational age [n (%)]					
Small	12	(26.7)	1	(1.1)	< 0.001
Appropriate	33	(73.3)	66	(73.3)	
Large	0	(0.0)	23	(25.6)	
During assessment	n	(%)	n	(%)	P
Monthly per capita family income (minimum salaries)					
? 0.25	15	(33.3)	26	(28.9)	0.65
0.26-0.50	19	(42.2)	46	(51.1)	
> 0.50	11	(24.4)	18	(20.0)	
Maternal schooling (years)					
1-8	11	(24.4)	32	(35.6)	0.27
³ 9	34	(75.6)	58	(64.4)	
Maternal age (years)					
? 19	4	(8.9)	11	(12.2)	0.77
> 19	41	(91.1)	79	(87.8)	
Number of people in household					
? 3	19	(42.2)	29	(32.2)	0.34
³ 4	26	(57.8)	61	(67.8)	
Children < 5 years					
1	34	(75.6)	65	(72.2)	0.84
2-5	11	(24.4)	25	(27.8)	
Sex					
Male	24	(53.3)	47	(52.2)	0.95
Female	21	(46.7)	43	(47.8)	
Age of infant (months)					
6-8	35	(77.8)	64	(71.1)	0.54
9-12	10	(22.2)	26	(28.9)	
Duration of exclusive breastfeeding (months)^§^					
? 2	7	(17.9)	13	(15.1)	0.89
³ 3	32	(82.1)	73	(84.9)	
Weight-for-age (Z-score)^||^					
< -1	11	(25.0)	6	(6.7)	0.007
³ -1	33	(75.0)	83	(93.3)	
Length-for-age (Z-score)^¶^					
< -1	17	(39.5)	15	(17.0)	0.009
³ -1	26	(60.5)	73	(83.0)	
Head circumference-for-age (Z-score)^*^					
< -1	5	(11.4)	7	(8.0)	0.54^**^
³ -1	39	(88.6)	80	(92.0)	

Missing cases: ^*^1 preterm/3 full-term, ^†^6 full-term, ^‡^Fisher’s exact test; ^§^10 infants were not exclusively breastfed; ^||^1 preterm/1 full-term; ^¶^2 preterm/2 full-term; SD = standard deviation.

Table 2 presents the mean cognitive, language and motor scores, which were all greater than 100, and no significant differences were found between the groups. For expressive communication, there was a mean difference of 0.7 points in the balanced score, to the detriment of the preterm infants (P = 0.06).

**Table 2.** Mean cognitive, language and motor scores among infants born preterm and full-term

Bayley III subtests	Preterm (n = 45)		Full-term (n = 90)		P
Composite scores	Mean	(SD)	Mean	(SD)	
Cognition	110.6	(9.1)	111.1	(7.6)	0.71
Language	108.2	(8.9)	109.7	(9.8)	0.39
Motor	106.1	(11.4)	108.1	(9.9)	0.30
Balanced scores					
Language					
Receptive communication	12.1	(2.0)	11.9	(2.1)	0.73
Expressive communication	10.6	(1.7)	11.3	(1.8)	0.06
Motor					
Fine motor skills	11.5	(2.4)	11.6	(1.8)	0.71
Gross motor skills	10.5	(2.5)	11.0	(2.4)	0.23

SD = standard deviation.

Table 3 shows the cognitive, language and motor scores according to socioeconomic, demographic, biological and nutritional variables. The girls had significantly higher mean scores in the three developmental domains. The duration of exclusive breastfeeding significantly influenced the language and motor domains, such that higher mean values were observed among the infants who breastfed for periods of three months or more. Poorer motor results were found among the infants who, at birth, had an Apgar score at 5 minutes of between 4 and 7 and a birth weight of less than 1500 g. The undernourished or nutritionally-at-risk infants showed lower motor performance than shown by the other infants, in relation to weight-for-age and length-for-age (< -1 Z score). A similar result was observed in relation to head circumference-for-age.

**Table 3.** Mean cognitive, language and motor scores according to socioeconomic, demographic, biological and nutritional variables

Variables	n =135	Developmental scores					
		Cognitive		Language		Motor	
		Mean	P	Mean	P	Mean	P
Monthly family per capita income (Minimum Salaries)							
? 0.25	41	109.1	0.22	108.1	0.32	109.1	0.43
0.26-0.50	65	111.9		109.1		106.3	
> 0.50	29	111.2		111.5		107.3	
Maternal schooling (years)							
1-8	43	110.5	0.65	108.8	0.75	106.0	0.30
³ 9	92	111.1		109.4		108.0	
Maternal age (years)							
? 19	15	111.3	0.84	112.4	0.16	108.2	0.76
> 19	120	110.9		108.8		107.3	
Number of people in household							
? 3	48	110.7	0.84	110.0	0.43	105.2	0.07
³ 4	87	111.3		108.7		108.6	
Children < 5 years							
1	99	110.7	0.69	109.6	0.38	106.9	0.38
2-5	36	111.4		108.0		108.7	
Sex							
Male	71	108.9	0.002	106.7	0.001	105.1	0.007
Female	64	113.2		111.9		109.9	
Birth weight (g)							
? 1499	14	107.1	0.24	106.3	0.41	100.8	0.04
1500-2499	28	112.1		109.4		109.1	
2500-2999	20	110.0		107.2		105.0	
³ 3000	73	111.4		110.1		108.7	
Size for gestational age							
Small	13	111.2	0.62	110.6	0.05	108.0	0.18
Appropriate	99	110.6		108.0		106.5	
Large	23	112.4		113.3		110.9	
Apgar score at 5 minutes^*^							
4-7	8	108.7	0.47	106.6	0.39	97.5	0.003
8-10	121	110.9		109.6		108.3	
Duration of exclusive breastfeeding (months)^†^							
? 2	20	108.7	0.18	105.2	0.02	102.8	0.02
³ 3	105	111.4		110.2		108.8	
Weight-for-age (Z-score)^‡^							
< -1	17	108.5	0.14	105.9	0.11	103.0	0.05
³ -1	116	111.5		109.8		108.2	
Length-for-age (Z-score)^§^							
< -1	32	110.9	0.84	107.0	0.13	104.3	0.03
³ -1	99	111.3		109.9		108.8	
Head circumference-for-age (Z-score)^§^							
< -1	12	110.0	0.59	104.4	0.07	101.1	0.02
³ -1	119	111.3		109.7		108.2	

Missing cases: ^*^6; ^†^10; ^‡^2 and ^§^4 infants were not exclusively breasdtfed.

## DISCUSSION

It has been shown in the literature that the first year of infants’ lives is the period of greatest development of functional capacity, with acquisition of skills in a number of different areas.^3^^,^^18^ Understanding the biopsychosocial factors that promote this development may enable interventions that are essential in developing countries, where infants are subject to a number of risk factors concerning their overall development. According to Grantham-McGregor et al.,^3^ it is highly probable that infants who do not achieve their full developmental potential will not go on to become fully productive adults.

Biological vulnerability caused by premature birth has been identified as one of the main risk factors for developmental delay.^4^^,^^5^^,^^6^ However, the complex interactions between biological and environmental factors throughout infants’ lives make it difficult to predict their development. Authors have gone on to emphasize that methodological issues have made it difficult to compare the results from several different studies: in other words, differences between the characteristics of infants in different studies make it impossible to extrapolate the results.^11^^,^^19^^,^^20^ Saigal and Doyle^11^ also stated that the definitions of developmental disorders, as well as the severity encountered, are not uniform, and that this would complicate comparisons. They observed that around 25% of surviving newborn preterm infants present significant neurological morbidity.

The results from the present study did not show any differences between the development of preterm and full-term infants, when age correction was used in the preterm group. The mean values reached by both groups in the cognition, language and motor domains were all considered normal in relation to the reference population. These data contrast with other reports that indicated differences in the development of preterm infants.^6^^,^^21^^,^^22^^,^^23^ However, some authors have claimed that simple comparisons should not be undertaken without considering the other innumerable variables that may influence development.^11^^,^^19^^,^^20^

A study by Lima et al.^4^ that was conducted in the same Brazilian state as the present one, using the Bayley-II scale on infants aged 12 months, found a difference between the groups of preterm and full-term infants, although the measurements obtained for the preterm infants were considered normal, i.e. without indicating any characteristics of delayed motor and/or mental development. On the other hand, results obtained by Woythaler et al.,^6^ using the Bayley scale on preterm infants of gestational age 34-37 weeks, found that the mean motor and mental values at 24 months were normal for the group as a whole. However, comparing them with full-term infants, they concluded that late preterm infants had poorer neurodevelopmental outcomes and increased odds of presenting delayed mental and/or physical development.

In southeastern Brazil, Mancini et al.^24^ used the Alberta Infant Motor Scale (AIMS) and did not observe any significant difference between preterm and full-term groups in relation to motor functions at 8 and 12 months of corrected age. Although the research tool was not the Bayley-II, it has been shown in the literature that there is a high correlation between the raw scores of the AIMS and the Bayley-II motor scale.^25^

In the present study, the findings in relation to expressive communication indicated that there was a tendency for preterm infants to present a poorer linguistic repertoire, in comparison with full-term infants, and this result was similar to what was described by Schirmer et al.^26^ Language developmental disorders are among the risks that prematurity may cause, and extreme prematurity tends to bring about even worse results, especially among males.^27^

In relation to the premature group in this sample, a number of important biological findings stand out: the mean gestational age of the group was 33 weeks, thus classifying the majority of the infants as moderately preterm, with a mean birth weight of 1762 g. The lowest Apgar score was 4, thus representing moderate to good birth vitality, and also confirming that the frequency of perinatal and postnatal morbidity was low. These findings place the group of preterm infants in a moderate risk category, and this needs to be highlighted, since greater caution is therefore required in generalizing the results.

Larroque et al.^23^ stated that the prevalence of disorders was greater among infants with a gestational age of less than 28 weeks, and that cognitive deficits were the most common finding in infants aged five years. This result leads to the idea that perhaps the age group of the present study (6-12 months) does not allow adequate inferences regarding cognitive issues, since studies have previously demonstrated that detection of subtle disorders was only possible in infants of preschool age.^12^^,^^27^

When investigating development, it is necessary to examine the context in which it occurs, especially the simultaneous presence of protective and risk factors. Certain aspects of the present study can be highlighted because of the possible protective action of development on preterm infants, such as the use of corrected chronological age, frequent practicing of exclusive breastfeeding, adequate perinatal care and regular maternal schooling.

The use of corrected chronological age is a strategy that provides protection for preterm infants against a possible erroneous diagnosis of delayed development: in other words, not using the corrected age would underestimate their performances in comparison with full-term infants. Several authors have advocated that corrected age should be used until the age of two years.^5^^,^^14^^,^^19^ In the present study, the chronological age was adjusted in accordance with the recommendations of the Bayley-III scales, as well as the clinical routine. However, some authors have questioned the possibility that performance might be underestimated.^14^^,^^19^^,^^28^ In this respect, Wilson and Cradock^28^ argued that there was only a real necessity for corrected chronological age for preterm infants born with less than 28 gestational weeks, low birth weight and/or additional perinatal and postnatal complications.

Exclusive breastfeeding throughout the whole sample was practiced by 92%, and among the premature infants, it was 87% (data not presented). Of these, 82% were exclusively breastfed for three months or more. According to Rey,^29^ breastfeeding offers additional advantages for preterm infants, since it helps with neurological development, especially cognition, by supplying the essential elements for nutrition and maturation of the brain and retina, as well as acting as a facilitator of mother-infant bonding.

Vohr et al.^30^ evaluated the impact of changes in perinatal care on neurodevelopment delay at 18 to 22 months of corrected age, among extremely low birth weight preterm infants with shorter gestation (22-26 weeks) and longer gestation (27-32 weeks) at three different times between 1993 and 1998. They observed that there was an improvement in low mental developmental indices, and also found that the survival rate increased over the course of this period. Administration of antenatal steroid was the only intervention associated with outcome improvement. Along these lines, it should be highlighted with regard to the present study that Hospital das Clínicas, which is part of UFPE, is a reference center specializing in attending high-risk pregnant mothers, and also participates in the Baby-Friendly Hospital Initiative, which proposes taking a more humane approach, and aims to support, encourage and protect breastfeeding practices.^31^

In the literature, maternal educational level is considered to be a moderating factor regarding infant development, since it favors infant care, concern for the importance of development and, consequently, improved quality of stimulation within the home.^6^^,^^9^ In a study by Stoelhorst et al.,^32^ it was observed that higher levels of maternal education were associated with improved mental development among premature infants aged between 18 and 24 months. It was confirmed in the present study that the mothers of preterm infants presented a moderate level of education, such that 76% had received secondary or higher education.

Considering that no developmental differences were observed, in this study, between the two groups, since infants in both groups had good cognitive, linguistic and motor responses, the possible influence of other variables on development was investigated throughout the whole sample. It was found that biological factors such as male sex, birth weight less than 1500 g, Apgar score £ 7 at 5 minutes after birth, weight-for-age, length-for-age and head circumference-for-age < -1 Z score and exclusive breastfeeding for less than or equal to two months were the factors that negatively influenced infants’ performance. This fact had already been observed by other authors, who reported that biological variables had a strong influence on the development of infants over the age range investigated.^2^^,^^3^^,^^4^

The infant’s sex was the only variable that showed cognitive, language and motor differences, such that the girls presented better performance than the boys. Other studies have reported similar results, thus indicating that females present better mental performance^4^^,^^18^ and that among very premature infants, males present much poorer mental and motor performance.^12^^,^^23^ Another important finding from the present study relates to motor development, which was found to be the domain most influenced by biological factors. This finding, as reported in the literature, emphasizes that motor development would seem to be more easily influenced by risk factors during the first years of life than mental development is.^18^

We should highlight that one of the limitations of the present study relates to the fact that a cross-sectional study design is not appropriate for detecting changes in biological and environmental factors that occur over time as developmental milestones. Another issue is the relatively small number of study subjects, which might have reduced the power to detect a difference in developmental scores between groups. Furthermore, the knowledge held by the researcher who performed the neurodevelopmental assessment on predictive variables (term and preterm neonates) may have caused information bias.

## CONCLUSIONS

Prematurity in itself had no impact on the development of the infants studied here, who were characterized as presenting moderate risk. However, it may have had an influence through several other factors resulting from these infants’ biological vulnerability and the possibility of unpleasant sensory motor experiences in the early stages of life. Thus, it is necessary to provide better healthcare and developmental surveillance during childhood to this group of infants, since impairments may occur in later life. Careful monitoring with the involvement of different healthcare professionals may be of great benefit, not only to the infants, but also to their families. Furthermore, research seeking better understanding of development among infants subjected to risk factors, especially those living in developing countries, needs to be encouraged.
